# Adult Triploid Rainbow Trout Can Adapt to Various Dietary Lipid Levels by Coordinating Metabolism in Different Tissues

**DOI:** 10.3390/metabo13030396

**Published:** 2023-03-08

**Authors:** Gege Liu, Lixia Chen, Haining Tian, Guoliang Sun, Fulei Wei, Yuqiong Meng, Rui Ma

**Affiliations:** 1 College of Ecological Environmental Engineering, Qinghai University, Xining 810016, China; 2 State Key Laboratory of Plateau Ecology and Agriculture, Qinghai University, Xining 810016, China

**Keywords:** rainbow trout, lipid, metabolism, liver, adipose tissue, muscle

## Abstract

Triploid rainbow trout can adapt to various dietary lipid levels; however, the mechanisms of systematic adaptation are not well understood. To investigate how adult triploid rainbow trout maintains lipid hemostasis under different exogenous lipid intake, a 77-day feeding trial was conducted. Diets with lipid contents of 20%, 25%, and 30% were formulated and fed to triploid rainbow trout with an initial weight of 3 ± 0.02 kg, and they were named L20, L25, and L30 group, respectively. Results showed that the condition factor, hepatosomatic index, liver color, and plasma triglyceride were comparable among three groups (*p* > 0.05), whereas the value of specific growth rate, viscerosomatic index, and liver glycogen content gradually increased with increasing dietary lipid level (*p* < 0.05). A significantly highest value of plasma glucose and nonesterified fatty acids were found in the L30 group (*p* < 0.05), whereas the significantly higher content of plasma total cholesterol, high-density lipoprotein–cholesterol, and low-density lipoprotein–cholesterol was found in the L25 group compared with those in L20 group (*p* < 0.05). As for lipid deposition, abdominal adipose tissue, and muscle were the main lipid storage place for triploid rainbow trout when tissues’ weight is taken into consideration. Overall quantitative PCR showed that the lipid transport and glycolysis were upregulated, and fatty acids oxidative was downregulated in liver when fish were fed low lipid diets. It meant that the liver was the primary lipid metabolizing organ to low lipid diet feeding, which could switch energy supply between glycolysis and fatty acids oxidation. Fish fed with a moderate dietary lipid level diet could increase lipid uptake and promote lipogenesis in muscle. Abdominal adipose tissue could efficiently uptake excess exogenous free fatty acid through upregulating fatty acid uptake and synthesis de novo and then storing it in the form of triglyceride. Excess lipid uptake is preferentially stored in abdominal adipose tissue through coordinated fatty acid uptake and fatty acid synthesis de novo as dietary lipid levels increased. In summary, triploid rainbow trout can adapt to various dietary lipid levels by coordinating metabolism in different tissues.

## 1. Introduction

Rainbow trout (*Oncorhynchus mykiss*) is one of the most widely cultured fish species in the world. Early sexual development in salmonids is a financial cost for aquaculture because metabolic energy is diverted from somatic growth to reproduction, resulting in a decline in appearance and fillet quality [1]. Therefore, rainbow trout with three sets of chromosomes (triploid rainbow trout) were produced to avoid gonads development. Due to their fast growth, delicious flesh, and high economic value, triploid rainbow trout have been widely cultured in China. Diets are essential for intensive fish farming. Dietary lipids play a vital role in fish growth, development, and metabolic processes. According to our previous study, subadult triploid rainbow trout can maintain lipid homeostasis by accumulating lipids in the muscle under various dietary lipid levels and tolerate high dietary lipid levels (up to 29.4%) with no negative effect on fish growth and liver health [2,3]. Few studies, or studies that only looked at specific organs, have been done on the impact of dietary lipids on lipid metabolism in adult triploid rainbow trout. Only one article in Nile tilapia (*Oreochromis niloticus*) reported the systematic adaptation of lipid metabolism through coordinating different tissues [4]. It is necessary to acquire additional knowledge about the systematic adaptation of adult triploid rainbow trout under various dietary lipid levels.

Increasing dietary lipid content generally improves lipid deposition in fish [5,6], and it is well known that excessive lipids are mainly stored in tissues including the liver, intestine, abdominal cavity, and muscle [7]. From the substance and energy metabolism point of view, the deposition of lipids in cells depends on lipid synthesis and lipolysis, which correlate with energy homeostasis [8]. Several key enzymes and transcriptional factors are involved in regulating these metabolic processes [9]. By hydrolyzing circulating triglycerides in the form of chylomicrons and very low-density lipoproteins, lipoprotein lipase (LPL) produces free fatty acids and 2-monoacylglycerols [10]. The free fatty acids were then taken up by the cluster of differentiation (*cd36*) [11] and transported intracellularly by fatty acid binding proteins (FABPs) [12]. Fatty acid synthetase (*fas*) is involved in fatty acid synthesis, whereas carnitine palmitoyl transferase 1 (*cpt1*) catalyzes the transport of long-chain fatty acids into the mitochondrial matrix, where they are required for the process of β-oxidation [13]. Being nuclear transcription factors, peroxisome proliferator-activated receptor (*ppar*) α, β/δ, and γ are believed to modulate lipid homeostasis by regulating the expression of the above genes [14]. Additionally, as neutral lipids are deposited as lipid droplets within cells, the accumulation and mobilization of lipid droplets are crucial in regulating lipid hemostasis from the perspective of subcellular structure. Lipid droplets are the universal cellular organelles for the transitory or long-term storage of lipids, consisting of a core of neutral lipids surrounded by a phospholipid monolayer (probably derived from the endoplasmic reticulum) and several dozens of associated proteins [15,16]. The perilipin family is the predominant structural protein of lipid droplets (LD), which sequester lipids by preventing lipase action on lipid droplets [17,18]. Long-lived proteins, LDs, or organelles that are no longer required can be destroyed via the evolutionarily conserved intracellular process known as autophagy for quick cell turnover [19,20]. The coordination of the above genes’ expression in different tissues has not been reported in triploid rainbow trout.

Therefore, the objective of the present study was to elucidate and clarify how adult triploid rainbow trout maintain lipid hemostasis in related tissues to adapt to various dietary lipid levels.

## 2. Materials and Methods

### 2.1. Fish Feed, Feeding, and Sampling

The current investigation was carried out in compliance with the Standard Operating Procedures of the Qinghai University Guide for the Use of Experimental Animals. A group of female triploid rainbow trout with an initial average weight of 3 ± 0.02 kg was obtained from Qinghai Minze Longyangxia Ecological Aquaculture Co., Ltd., China. We evenly divired 4500 fish into nine net cages (8 m × 8 m × 8 m). Three isonitrogenous experimental diets (dietary protein level: 38% dry matter) with graded lipid levels of 20, 25, and 30% were formulated as described in [2], and they were named L20, L25, and L30 groups, respectively. Diet formulation and proximate compositions of the experimental diets are shown in Table 1. Each diet was randomly fed three net cages for 77 days. The water temperature stayed between 8 and 16 °C, and the dissolved oxygen concentration exceeded 7 mg/L. At the end of the feeding trial, all the experimental fish fasted for 3 days. One fish per net cage was stored at −20 °C for the determination of whole-body lipid content. Six fish per net cage was randomly selected and anesthetized with eugenol (1: 10,000) (Shanghai Reagent Corp, Shanghai, China). After the weight of the body, viscera, and liver were recorded, and the length of fish was measured, liver, abdominal adipose tissue, and dorsal muscle samples were taken and kept in liquid nitrogen, then stored at −80 °C for later analysis. Blood collection from the caudal vein, and sampled blood were kept at 4 °C; the serum was separated by centrifugation (3000 rpm for 10 min) at 4 °C and stored in liquid nitrogen before being frozen at −80 °C. The liver samples of three fish per cage were preserved with 4% paraformaldehyde for morphological analysis.

### 2.2. Chemical Analysis

The plasma glucose, triglyceride, total cholesterol, higher-density lipoprotein cholesterol (HDL-C), and low-density lipoprotein cholesterol (LDL-C) were assayed the same as described in [21]. Plasma non-esterified fatty acids (NEFA) were determined using commercial kits (Jiancheng Bioengineering Institute, Nanjing, China) according to the manufacturer’s protocols. As previously reported [22], the lipid content of whole fish, abdominal adipose tissue, liver, and dorsal muscle was extracted using chloroform: methanol (2:1, *v*/*v*).

### 2.3. Hepatic Morphology Analysis

The paraffin sections were prepared using normal histological procedures. Briefly, they were transformed into paraffin wax blocks, sectioned to a thickness of 6 μm using a microtome, and stained with hematoxylin and eosin (HE) or periodic acid-Schiff (PAS) for examination by light microscopy (ECLIPSE NieU, Nikon Corporation, Tokyo, Japan). For frozen sections, another sample was randomly selected from each cage and sliced with a freezing microtome into 7-μm-thick sections that were dyed with oil red (OR).

### 2.4. qRT-PCR Analysis of Lipid-Related Genes

RNA isolation, cDNA synthesis, and qPCR were performed as described previously [3]. RNA simple total RNA Kit (TIANGEN, China) was used to isolate total RNA from the liver, abdominal adipose tissue, and muscle of triploid rainbow trout according to the manufacturer’s protocol. RNA quality and quantity were determined by measuring absorbance at 260 nm and 280 nm. RNA was reverse transcribed into cDNA using the PrimeScriptTM RT reagent Kit (Takara, Japan).

The mRNA expression levels of the genes in the liver, abdominal adipose tissue, and muscle were assayed by quantitative RT-PCR using the TB GREEN^®^ *Premix Ex Taq*^TM^II (Tli RNaseH Plus) Kit (Takara, Japan). PCR reactions of 10 μL final volume contained 1 μL of cDNA, 0.3 μL (10 μM) of forward and backward primer, 5 μL of PCRTaqMix (2×), and 3.4 μL of ddH_2_O. The PCR process was performed as follows: 95 °C for 5 min, 95 °C for 5 s, and 60 °C for 30 s, followed by 72 °C for 1 s. The relative mRNA expression levels of target genes were calculated by the 2^−ΔΔCt^ method.

The genes investigated in this study were related to lipid uptake (lipoprotein lipase [*lpl*], cluster of differentiation 36 [*cd36*], fatty acid transport protein 1 [*fatp1*], fatty acid transport protein 5 [*fatp5*]); intracellular fatty acid transportation (fatty acid binding protein 3 [*fabp3*], fatty acid binding protein 4 [*fabp4*]); fatty acid biosynthesis de novo (fatty acid synthase [*fas*], acetyl-CoA carboxylase α [*accα*]); fatty acid β-oxidation (carnitine palmitoyl transferase 1 [*cpt1*], acetyl-CoA oxidase [*aco*]); metabolic regulatory factors (peroxisome proliferator-activated receptor α [*pparα*], peroxisome proliferator-activated receptorβ [*pparβ*], peroxisome proliferator-activated receptorγ [*pparγ*] and sterol regulatory element binding protein 1 [*srebp1*]); very low-density lipoprotein related protein (apoprotein B100 [*apoB100*] and apoprotein E [*apoE*]); lipid droplet metabolism (perilipin1 [*plin1*], perilipin3 [*plin3*]) and autophagy (autophagy-related gene 7 [*atg7*], autophagy-related gene 12 [*atg12*]); carbohydrate metabolism (glucokinase [*gk*], pyruvate kinase [*pk*], glucose-6-phosphatase [*g6pase*], and phosphoenol pyruvate carboxykinase [*pepck*]). Specific primer of the genes was designed referring to the relevant cDNA sequences of rainbow trout listed in Table 2.

### 2.5. Calculations

Specific growth rate (SGR, %/day) = 100 × ln [final body weight (g)/initial body weight (g)]/days of the experiment;

Condition factor (CF) = 100 × [body weight (g)]/[body length (cm)]^3^;

Viscerosomatic index (VSI, %) = 100 × [viscera weight (g)]/[body weight (g)];

Hepatosomatic index (HSI, %) = 100 × [hepatic weight (g)]/[body weight (g)].

### 2.6. Statistical Analysis

Data were assessed by one-way ANOVA using SPSS 25.0 for windows. When differences were significant (*p* < 0.05), Tukey’s test was used to compare the means among individual treatments.

## 3. Results

### 3.1. Growth Performance, Organ Indexes, and Plasma Biochemistry

As shown in Table 3, fish from the L30 group had significantly higher values of SGR than the L20 and L25 groups (*p* < 0.05). No significant difference was found in CF, HSI, and liver color (*p* > 0.05). As the level of dietary lipid increased, VSI gradually increased with a significantly higher value observed in the L30 group compared with the L20 group (*p* < 0.05). The liver glycogen content significantly increased as dietary lipid content increased from 20% to 30% (*p* < 0.05).

No significant difference was found in plasma triglyceride (*p* > 0.05). A significantly higher value of plasma glucose and NEFA were found in the L30 group compared to L20 and L25 groups (*p* < 0.05), respectively. A significantly higher content of plasma total cholesterol, HDL-C, and LDL-C was found in the L25 group compared with those in the L20 group (*p* < 0.05).

### 3.2. Lipid Deposition

The lipid content of whole fish, abdominal adipose tissue, liver, and muscle tissues are shown in Table 4. The significantly higher value of whole fish lipid content (calculated as g/100 g tissue wet weight) was observed in L25 and L30 groups (*p* < 0.05). As dietary lipid content increased, the lipid content of abdominal adipose tissue and muscle gradually increased and reached their maximum average value in the L30 group (*p* < 0.05). No significant difference was observed for hepatic lipid content (*p* > 0.05). When the weight of tissues and the whole body were considered (calculated as g/tissue of a 100 g fish), the lipid content of muscle gradually increased as dietary lipid increased (*p* < 0.05), the significantly highest value of abdominal adipose tissue lipid content was found in the L30 group (*p* < 0.05). No significant difference in hepatic lipid content was observed (*p* > 0.05).

### 3.3. The Histological Appearance of Liver

**Figure 1:**
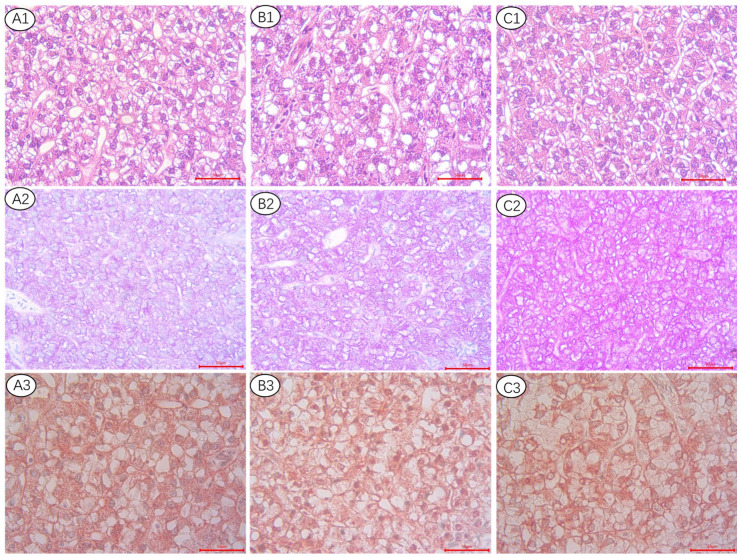
Histological appearance of liver from triploid rainbow trout fed the 20% lipid content (**A1**–**A3**), 25% lipid content (**B1**–B**3**), and 30% lipid content (**C1–C3**) diet, respectively. Hematoxylin-eosin (HE) staining: (**A1**–**C1**). Magnification ×400. Periodic acid-Schiff (PAS) staining: (**A2**–**C2**), Magnification ×400. Oil red staining: (**A3**–**C3**), Magnification ×400.

As shown in Figure 1, the hepatocellular structure of all treatments seems not obviously different by using HE staining (A1, B1, C1). By applying PAS (A2, B2, C2) and OR (A3, B3, C3) staining, glycogen and lipid droplets were stained with purple and red color, respectively. With increasing dietary lipid levels, liver glycogen staining deepens in purple, whereas OR staining found no significant changes in lipid droplet size or quantity.

### 3.4. The Relative mRNA Expression Levels of Hepatic Metabolism Genes

**Figure 2:**
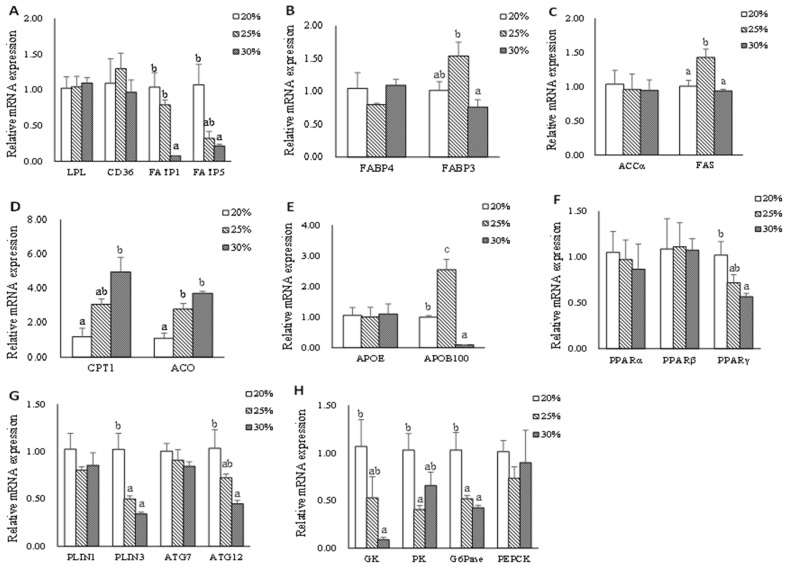
The mRNA expression of genes related to lipid metabolism in hepatic of triploid rainbow trout fed with diets containing low (20%), medium (25%), and high (30%) lipid content for 77 days. (**A**) The relative mRNA abundance of *lpl*, *cd36*, *fatp1,* and *fatp5* shows the ability of lipid uptake. (**B**) The relative mRNA abundance of *fabp3* and *fabp4* shows the activity of intracellular FA transport. (**C**) The relative mRNA abundance of accα and fas shows the FA biosynthetic activity de novo. (**D**) The relative mRNA abundance of *cpt1* and *aco* shows the activity of FA β-oxidation. (**E**) The relative mRNA abundance of *apoE* and *apoB100* which are important components of VLDL. (**F**) The relative mRNA abundance of *pparα*, *pparβ,* and *pparγ* in the liver. (**G**) The relative mRNA abundance of *plin1*, *plin3*, *atg7,* and *atg12* in the liver. (**H**) The relative mRNA abundance of *gk*, *pk*, *G6Pase,* and *pepck* in the liver. Values are expressed as the means ± S.E.M. (n = 3). Statistical significance was evaluated using one-way ANOVA, followed by Tukey’s test. Values with different letters on columns statistically differ at *p* < 0.05.

Data on hepatic mRNA expression of genes related to lipid metabolism and glycometabolics are shown in Figure 2. The mRNA expression of *fatp5* and *fatp1* genes were down-regulated in the L30 group compared with that in the L20 group (*p* < 0.05). However, there were no significant differences in the mRNA expression levels of *lpl* and *cd36* genes (*p* > 0.05). Dietary lipid level did not significantly influence the gene expression of *fabp4*, *accα,* and *apoE* (*p* > 0.05); however, the significantly higher gene expression of *fabp3* and *fas* were found in the L25 group compared with that in the L30 group (*p* < 0.05). The hepatic mRNA expression levels of *cpt1* and *aco* genes increased in fish-fed diets with high dietary lipid levels (*p* < 0.05). The significantly highest gene expression of *apoB100* was found in the L25 group compared with that in L20 and L30 groups (*p* < 0.05). There were no significant differences in the hepatic mRNA levels of *pparα*, *pparβ*, *plin1*, and *atg7* (*p* > 0.05). A significantly higher expression level of *plin3* was observed in the L20 group (*p* < 0.05). The gene expression of *pparγ* and *atg12* decreased as dietary lipid levels increased (*p* < 0.05). The significantly higher expression levels of *gk*, *pk,* and *G6pase* genes were observed in the L20 group (*p* < 0.05). There was no significant difference in the mRNA expression levels of *pepck* (*p* > 0.05).

### 3.5. The Relative mRNA Expression Levels of Abdominal Adipose Tissue Metabolism Genes

**Figure 3:**
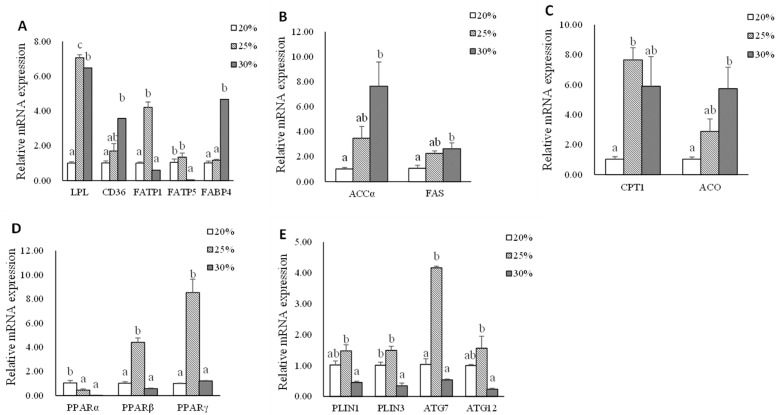
The mRNA expression of genes related to lipid metabolism in abdominal adipose tissue of triploid rainbow trout fed with diets containing low (20%), medium (25%), and high (30%) lipid content for 77 days. (**A**) The relative mRNA abundance of *lpl*, *cd36*, *fatp1*, *fatp5,* and *fabp4* shows the ability of lipid uptake. (**B**) The relative mRNA abundance of *accα* and *fas* shows the FA biosynthetic activity de novo. (**C**) The relative mRNA abundance of *cpt1* and *aco* shows the activity of FA β-oxidation. (**D**) The relative mRNA abundance of *pparα*, *pparβ,* and *pparγ* in adipose tissue. (**E**) The relative mRNA abundance of *plin1*, *plin 3*, *atg7,* and *atg12* in adipose tissue. Values are expressed as the means ± S.E.M. (n = 3). Statistical significance was evaluated using one-way ANOVA, followed by Tukey’s test. Values with different letters on columns statistically differ at *p* < 0.05.

Data on adipose tissue mRNA expression of genes related to lipid metabolism are shown in Figure 3. The significantly highest gene expression of *lpl* was found in the L25 group compared with that in L20 and L30 groups (*p* < 0.05). The mRNA expressions of *cd36* and *fabp4* were up-regulated (*p* < 0.05). The significantly higher gene expression of *fatp1* was found in the L25 group compared with that in L20 and L30 groups (*p* < 0.05). However, the significantly higher gene expression of *fatp5* was found in L20 and L25 groups compared with that in the L30 group (*p* < 0.05). The mRNA expression levels of *accα*, *fas,* and *aco* genes in the L30 group were significantly higher than the L20 group (*p* < 0.05). The mRNA expression of the *cpt1* gene was up-regulated in the L25 group compared with that in the L20 group (*p* < 0.05). The significantly highest gene expression of *pparβ* and *pparγ* was found in the L25 group, whereas the significantly highest gene expression of the *pparα* gene was found in the L20 group (*p* < 0.05). The significantly higher expression levels of *plin1*, *plin3*, *atg7,* and *atg12* genes were observed in the L25 group (*p* < 0.05).

### 3.6. The Relative mRNA Expression Levels of Muscle Metabolism Genes

**Figure 4:**
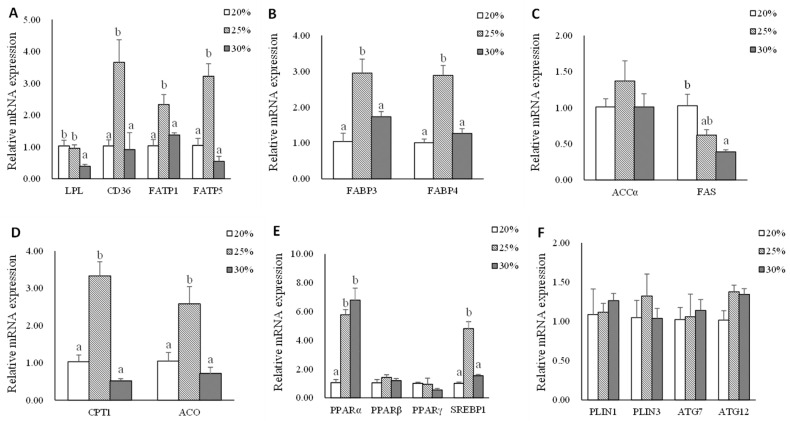
The mRNA expression of genes related to lipid metabolism in muscle of triploid rainbow trout fed with diets containing low (20%), medium (25%), and high (30%) lipid content for 77 days. (**A**) The relative mRNA abundance of *lpl*, *cd36*, *fatp1*, and *fatp5* shows the ability of lipid uptake. (**B**) The relative mRNA abundance of *fabp3* and *fabp4* shows the activity of intracellular FA transport. (**C**) The relative mRNA abundance of *accα* and *fas* shows the FA biosynthetic activity de novo. (**D**) The relative mRNA abundance of *cpt1* and *aco* shows the activity of FA β-oxidation. (**E**) The relative mRNA abundance of *pparα*, *pparβ*, *pparγ*, and *srebp1* in muscle. (**F**) The relative mRNA abundance of *plin1*, *plin3*, *atg7*, and *atg12* in muscle. Values are expressed as the means ± S.E.M. (n = 3). Statistical significance was evaluated using one-way ANOVA, followed by Tukey’s test. Values with different letters on columns statistically differ at *p* < 0.05.

Data on muscle mRNA expression of genes related to lipid metabolism are shown in Figure 4. The significantly higher gene expression of *lpl* was found in L20 and L25 groups compared with that in the L30 group (*p* < 0.05). The mRNA expression levels of *cd36*, *fatp1*, *fatp5*, *fabp3*, *fabp4*, *cpt1*, *aco,* and *srebp1* genes in L25 group were significantly higher than L20 and L30 groups (*p* < 0.05). There were no significant differences in the mRNA expression levels of the *accα* gene in the muscle of triploid rainbow trout (*p* > 0.05). The mRNA expression of *fas* gene was down-regulated as dietary lipid level increased (*p* < 0.05). The significantly higher gene expression of *pparα* was found in L25 and L30 groups compared with that in the L20 group (*p* < 0.05). There were no significant differences in the mRNA expression levels of *pparβ*, *pparγ*, *plin1*, *plin3*, *atg7,* and *atg12* (*p* > 0.05).

## 4. Discussion

In this study, triploid rainbow trout grew from 3 to 4 kg during 77 days. At the end of the trial, fish from the L30 group had a significantly higher value of specific growth rate (SGR) than the L20 group (*p* < 0.05). This was in accordance with our previous study on subadult triploid rainbow trout [2]. Although there was no significant difference for CF in the present study, the upward trend of VSI and lipid content of whole fish was consistent. A previous study in rainbow trout also found that VSI and final whole-body lipid content was positively related to dietary lipid levels and increased lipid deposition in the visceral cavity of larger trout mostly due to a hypertrophic response [5]. No significant changes in the hepatosomatic index (HSI), liver color, and hepatic lipid content were observed among the three groups, which suggested that adult triploid rainbow trout could maintain hepatic lipid homeostasis and therefore, tolerate dietary lipid levels (varied from 20% to 30%). Similar findings in our previous study showed that subadult triploid rainbow trout could maintain hepatic lipid homeostasis when fed diets with lipid levels ranging from 14.8% to 29.4% [3]. When it comes to lipid synthesis and storage, visceral adipose tissue and the liver of fish are both important organs that play a role in maintaining lipid homeostasis and energy balance [23,24,25]. Thus, we further analyzed the lipid content of different tissues. The results of the study found that the lipid content of abdominal adipose tissue and muscle gradually increased and reached their maximum average value in the L30 group. When the weight of tissues and the whole body were evaluated (measured as g/tissue of a 100 g fish), the muscle tissue (average value ranged from 7.8–8.59 g/tissue of a 100 g fish) contributes slightly more to the lipid deposition than abdominal adipose tissue (average value ranged from 6.57–8.07 g/tissue of a 100 g fish). This is different from subadult rainbow trout, in which was found that the muscle tissue (average value ranged from 5.64–6.74 g/tissue of a 100 g fish) contributes equally as visceral tissue (average value ranged from 4.97–6.42 g/tissue of a 100 g fish) to the lipid accumulation from the whole fish perspective. Although both abdominal adipose tissue and muscle are the main lipid deposition sites for triploid rainbow trout, adult triploid rainbow trout seemed to prefer to accumulate lipids in the muscle. A similar study on another fatty fish, Atlantic salmon (*Salmo salar*) largely confirmed these findings, Bjerkeng et al. [26] observed that fish fed high lipid levels diets obtained higher lipid content of total carcass lipid, abdomen, and myosepta.

The liver, which plays a crucial role in regulating lipid metabolism, uptakes and processes chylomicron remnants and actively produces cholesterol, bile acids, and fatty acids during the postprandial period. The esterified fatty acids are then stored in lipid droplets or released as VLDL [27,28,29]. To explore the mechanism of rainbow trout adapting to various lipid levels, we further examined the key gene expression of lipid metabolism. Significantly higher fatty acid transport (*fatp1* and *fatp5*) and lower fatty acids (FA) oxidation gene (*cpt1* and *aco*) expression were observed in the L20 group, which suggested that rainbow trout increased lipid transport and decreased fatty acids oxidative energy supply to counter the potential negative effects of limited exogenous lipid. This might be related to the higher mRNA expression of *pparγ* in the L20 group. By specifically regulating the expression of the genes for *fatp1* and *cd36*, *pparγ* regulates adipogenesis and keeps the balance between glucose and lipid oxidation [30]. Accordingly, glycolysis (*gk*, *pk*) was also upregulated in the L20 group. This is similar to our previous results on subadult rainbow trout, which suggested that rainbow trout could switch energy supply between glycolysis and fatty acids oxidation [3]. The results of the study also found that liver glycogen content significantly increased with increasing dietary lipid levels, which was consistent with the histomorphology reflection of the liver. LD-associated proteins perilipin (plin) are crucial for both adipogenesis and lipolysis [31]. Perilipin 3 covers nascent neutral lipids and affects the ability of the cell to accumulate TG [32,33]. Autophagy is crucial for basal homeostasis and is associated with the energy balance and nutritional state of cells [34]. This process is carried out by autophagy-related genes (ATGs), which mobilize lipid droplets to provide fatty acids for mitochondrial oxidation [35]. In the present study, the mRNA expression of lipid droplet mobilization (*plin3*) and autophagy (*atg12*) genes were upregulated in the L20 group. This suggests that when exogenous lipid intake is limited, the liver regulates lipid droplet mobilization and autophagy to maintain intracellular lipid homeostasis. Consistently, hepatic OR staining showed that the size or number of lipid droplets had no significant changes among the three groups suggesting that triploid rainbow trout had adaptive mechanisms to maintain lipid homeostasis in the liver. Adipose and muscle tissues reacted similarly when fish were fed an L20 diet, both adapted to low lipid intake by down regulated lipid uptake, transport, and β-oxidation. The plasma TG content was kept constant as a result of lipid homeostasis. Therefore, fish fed with a low lipid diet could increase FA uptake and accelerate the utilization of carbohydrates to satisfy the physiological requirement of lipid utilization, and this process is mainly performed in the liver.

When fish were fed with moderate dietary lipid levels, the liver reacted actively by upregulated lipid transportation (*fabp3*), de novo synthesis (*fas*), and VLDL assembly (*apoB100*), which was in accordance with the trend of plasma total cholesterol, HDL-C, and LDL-C. These results agree well with the finding in Nile tilapia *(Oreochromis niloticus*) [36] and Turbot (*Scophthalmus maximus* L.) [37]. According to earlier research, plasma apolipoproteins influence the transport and redistribution of lipids among tissues and cells as well as lipoprotein metabolism [38]. The high expression of hepatic lipoprotein in the L25 group suggested that freshly produced TG may also be delivered efficiently to peripheral tissues for further utilization. The previous study showed that *pparγ* slows down the rate of lipolysis, principally in adipose tissue [39,40], and *pparβ* is found at high levels in many tissues and increases the oxidation of fatty acids in skeletal muscle and adipose tissue [41,42]. The present results also found that adipose tissue in the L25 group upregulated the expression of *pparβ* and *pparγ*, therefore, stimulating lipid uptake (*lpl* and *fatp1*), fatty acid β-oxidation (*cpt1*), and lipid droplet (*plin1* and *plin3*), indicating that adipose tissue reacted actively when fish were fed diets with moderate dietary lipid levels. It was interesting that autophagy (*atg7* and *atg12*) was also stimulated in the L25 group. By searching the literature, a previous transcriptomic analysis reported that rainbow trout preadipocytes upregulated autophagy-related genes expression during the adipogenic process in vitro (proliferation and differentiation phases) [43], suggesting a possible role of autophagy mediators in fish adipogenesis. Moreover, the higher mRNA expression of lipid uptake (*cd36*, *fatp1,* and *fatp5*), fatty acids transport (*fabp3* and *fabp4*), and fatty acids β-oxidation (*cpt1* and *aco*) of muscle tissue in the L25 group indicated that the muscle of triploid rainbow trout fed with the moderate dietary lipid level diet was highly probable to be stimulated. *Srebp1* plays important role in lipogenesis [44]. The significantly highest gene expression of *srebp1* was found in the L25 group and promised that the FA would be efficiently synthesized and esterified to form TG in muscle. Fish fed an L25 diet could increase lipid uptake and promote lipogenesis in the muscle which is in line with the increased lipid content in the muscle of the fish.

When fish were fed with a high lipid diet, significantly lower lipid uptake (*fatp1*, *fatp5,* and *fabp3*) and higher fatty acids oxidation gene (*cpt1* and *aco*) expression in the liver were observed in the L30 group, which suggested that rainbow trout decreased lipid uptake and increased fatty acids oxidation to adapt excess exogenous lipid intake. The results of the study also found that the mRNA expressions of *plin3*, *atg12*, *gk*, *pk,* and *apoB100* were downregulated. The possible reason was that the liver regulated the source of energy supply, reduced lipoprotein secretion, and lipid deposition to maintain lipid homeostasis when fish were fed a diet with high dietary lipid levels. Additionally, the higher mRNA expression of the genes related to exogenous FA uptake (*cd36*, *lpl*), FA transport (*fabp4*), and FA synthesis de novo (*accα* and *fas*) in the L30 groups indicated that abdominal adipose tissue could efficiently uptake excess exogenous FFA and store it in the form of TG. In a previous study on Nile tilapia (*Oreochromis niloticus*), it was determined that diet-derived FFAs are mostly absorbed by adipocytes, and that in the presence of a high lipid intake, the FA synthesis de novo also increases [4]. FA oxidation-related genes, such as *cpt1* and *aco*, were also expressed higher in the L30 group, suggesting that excess TG in abdominal adipose tissue would be lipolyzed to limit the capacity for lipid accumulation. According to our findings, the principal sites for lipid deposition in adult triploid rainbow trout are abdominal adipose tissue and muscle. Both of these tissues’ lipid contents showed an upward trend with increasing dietary lipid levels. However, it was noticed that the muscle *lpl* mRNA expression was down-regulated with increasing dietary lipid levels in the present study. A different result was reported by Dong et al. [45], who observed the mRNA expression *lpl* gene was up-regulated of grass carp (*Ctenopharyngodon idella*) of increasing conjugated linoleic acid diet intake. The differences in these results may arise from the discrepancy in lipid tolerance in different fish species. Additionally, a significantly lower expression level of the *fas* gene was observed in the L30 group. This may be related to the high content of fish oil which is rich in n-3 long-chain polyunsaturated fatty acid (n-3LC PUFA), especially EPA (20: 5n-3) and DHA (22: 6n-3) in high lipid diet [46]. Through broad modulation of lipid metabolism by blocking lipogenesis, the n-3 PUFA primarily exerts its fat-reducing impact [47]. PPARα, which is a subtype of the PPAR subfamily, is a key regulator of peroxisomal and mitochondrial β-oxidation of fatty acids, ketone body synthesis, and systemic lipid metabolism that can regulate lipolysis [48,49]. The mRNA expression of the PPAR*α* gene was up-regulated when the dietary lipid level increased. However, the mRNA levels of some *pparα*-targeted genes in FA β-oxidation, such as *cpt1* and *aco*, had no significant difference compared to the L20 group. The potential cause was the discrepancy between mRNA levels, protein levels/activities, and metabolic flux. These results suggested that the abdominal adipose tissue of triploid rainbow trout is more likely to be the primary organ that responds to a high lipid intake than the muscle.

In conclusion, when lipid intake is limited, the liver counters the negative effects by increasing lipid uptake and switching the energy supply between glycolysis and fatty acid oxidation. In contrast, excess lipid uptake is preferentially stored in abdominal adipose tissue through coordinated FA uptake and FA synthesis de novo as dietary lipid levels increased. Triploid rainbow trout can adapt to various lipid levels by coordinating metabolism in different tissues.

## Figures and Tables

**Table 1:** Formulation and proximate compositions of the experimental diets (% dry matter).

Ingredients	Diets1 (20%)	Diets2 (25%)	Diets3 (30%)
Brown fish meal ^1^	51	51	51
Wheat meal ^1^	15	15	15
Cassava starch ^1^	5	5	5
Cellulose	11.36	6.36	1.36
Fish oil	11.8	16.8	21.8
Soybean oil	3	3	3
Vitamin-mineral premix ^2^	1	1	1
Ca (H_2_PO_4_)_2_	0.8	0.8	0.8
Choline chloride	0.3	0.3	0.3
Calcium propionate	0.1	0.1	0.1
Ethoxyquin	0.05	0.05	0.05
Betaine	0.5	0.5	0.5
Y_2_O_3_	0.05	0.05	0.05
Astaxanthin ^3^	0.04	0.04	0.04
Proximate analysis (n = 3, % dry matter)			
Crude protein	40.6	39.7	39.9
Crude lipid	19.8	26.1	30.5
Gross energy (KJ/g)	16.3	18.5	20.2
P/E ratio (mg protein/KJ)	24.9	21.4	19.7

^1^ Brown fish meal (Origin: Peru; Produced by Corporacion pesquera inca s.a.c.), crude protein 67.1%, crude lipid 8.5%; Wheat meal (Origin: China; Produced by Shanxi bull flour Co., Ltd., Baoji, China), crude protein 16.5%, crude lipid 2.6%; Cassava starch (Origin: China; Produced by Xiamen Xiangyu agricultural Co., Ltd., Xiamen, China), crude protein 0.83%, crude lipid 0.08%. ^2^ Vitamin–mineral premix included 0.5% vitamin premix and 0.5% mineral premix (Carnivorous Fish Formula provided by Tongwei Co., Ltd., Chengdu, China). Vitamin premix included the following (each kg^−1^ diet): vitamin A, 50 KIU; vitamin D3, 20 KIU; vitamin E, 390 mg; vitamin K, 150 mg; vitamin B1, 120 mg; vitamin B2, 165 mg; vitamin B6, 130 mg; vitamin B12, 0.5 mg; biotin, 2.4 mg; folic acid, 75 mg; inositol, 1200 mg; niacin, 670 mg; ascorbic acid, 2500 mg. Minerals premix included the following (mg kg^−1^ diet): sodium, 1500; iron, 3000; copper, 90; zinc, 1500; manganese, 800; selenium, 4.3; iodine, 21; cobalt, 3. ^3^ Astaxanthin: 10% (CAROPHYLL^®^, DSM, Heerlen, The Netherlands).

**Table 2:** Primer sequences for real-time quantitative PCR.

Gene Symbol	Gene Name	Primer Sequence (5′-3′)		Genbank Accession No.
*Reference gene*				
*β-actin*	β-actin	F:TACAACGAGCTGAGGGTGGC	R: GGCAGGGGTGTTGAAGGTCT	AJ438158.1
*Lipid uptake*				
*lpl*	Lipoprotein lipase	F:GGACGTTGGGGAGCTGCTTA	R: ATTGAGTCTCCCCGGCCTTG	NM_001124604.1
*cd36*	Cluster of differentiation 36	F:GTCGTGGCTCAAGTCTTCCA	R: TCAAATACTCGGCTCGCCTC	AY606034.1
*fatp1*	Fatty acid transport protein 1	F:GTCCCGTGTTCCTACGCATCT	R:GCCTCATAACGCCCTGCTCT	XM_036941441.1
*fatp5*	Fatty acid transport protein 5	F:TGGGAAGACTTTTGATGGGC	R:CTGGATACGGATGAAATGAGGT	AF072760.1
*fabp3*	Fatty acid binding protein 3	F:ATGGCTGAGGCATTCG	R:CATCTTACCACCGTCTATC	NM_001124713.1
*fabp4*	Fatty acid binding protein 4	F:CGTTGGAACTTGGAAGATGACT	R:TGCCGAGTAGCAAAACCCA	XM 021584874.2
*Triacylglycerol synthesis and catabolism*				
*accα*	Acetyl-CoA carboxylase α	F:CCCATTCGCCTTTTCCTTAC	R:ACCCTGCGTGTCCCCATA	XM_021623129.1
*fas*	Fatty acid synthase	F:TCTAGAGACGCCACCTTCGA	R:TGCAGTTTCTCCTCAGCCAG	XM_021581290.1
*cpt1*	Carnitine palmitoyl transferase 1	F:TACAGCTGGCCCAATTCAGG	R:TCGCAGTGTTCTTGTCCTCC	AF327058.3
*aco*	Acetyl-CoA oxidase	F:TTGGGCCTCATCATTGCAGT	R:ACTGGGTCTGGTGCTCAATG	XM_021613038.1
*Very low-density lipoprotein protein*				
*apoE*	apolipoprotein E	F:ACCTGGGAGAGCTCAAGACT	R:CACTGTGTTGCGGATCTCCT	NM_001124346.1
*apoB100*	Apolipoprotein B100	F:GTCTGCCACCATGTTCTCCA	R:CTGGATGGCCTGCTCAAGAA	XM_ 021611526. 1
*Transcription factors*				
*pparα*	Peroxisome proliferator-activated receptor α	F:CACTCCACCCTTCGTCATCC	R:CCTCAGCCTCCTTCTCAAGC	HM536190.1
*pparβ*	Peroxisome proliferator-activated receptor β	F:GGGGTACACGTGTTCTACCG	R:GTACTTCAGCAGCGTCACCT	HM536191.1
*pparγ*	Peroxisome proliferator-activated Receptor γ	F:GAGCTGGACATGAACGACCA	R:TGTGCCGTCCTTGTTCATGA	NM_001197212.1
*srebp1*	Sterol regulatory element-binding protein 1	F:TCCTCTCCCTCAATCCCCTG	R:CGAGTCAGCTGCGTTGTCT	KP342261.1
*lipid droplets metabolism*				
*plin1*	Perilipin1	F:AAGGTCAGGAACTGGTCACAC	R:TCTGAGGACTGTGCTGTTGTC	XM_021598341.2
*plin3*	Perilipin3	F:GAGAAGGGAGAGGACCTGGA	R:CCTGGGAGACCCTGTACACT	XM_021603908.2
*atg7*	autophagy-related gene 7	F:TACTGAAGGAGGTTATGCG	R:TGATCTGATGAGGGACGAG	XM_036984267.1
*atg12*	autophagy-related gene 12	F:TGGAGGCCAATGAACAGCTG	R:CTTCCCATCGCTGCCAAAAC	XM_021623074.1
*Carbohydrate metabolism*				
*gk*	Glucokinase	F:AGATCACTGTGGGCATCGAC	R:GATGTCACAGTGAGGCGTCA	AF053331.2
*pk*	Pyruvate kinase	F:GTTCCCTGTCGAGTCTGTGG	R:CAGACGACGAAGCTCCTCAA	XM_021622264.1
*g6pase*	Glucose-6-phosphatase	F:GCTGACCTGCATACCACCTT	R:CAGCCACCCAGATGAGCTTT	XM_021575943.1
*pepck*	Phosphoenol pyruvate carboxykinase	F:CGGTGTGTTTGTAGGAGCCT	R:ACGTGGAAGATCTTGGGCAG	NM_001124275.1

**Table 3:** Growth performance, organ indexes, and lipid content of whole fish, abdominal adipose tissue, liver, and muscle of triploid rainbow trout fed the experimental diets for 77 days ^1^.

	Dietary Lipid Levels (% Dry Matter)			F	*p*
	20	25	30		
Growth performance					
SGR ^2^	0.37 ± 0.01 ^a^	0.42 ± 0.02 ^ab^	0.43 ± 0.01 ^b^	6.91	0.03
Organ indexes					
Condition factor ^3^	2.15 ± 0.04	2.19 ± 0.04	2.12 ± 0.03	0.93	0.42
Viscerosomatic index (%) ^4^	13.27 ± 0.13 ^a^	13.91 ± 0.76 ^ab^	15.21 ± 0.16 ^b^	4.75	0.03
Hepatosomatic index (%) ^5^	1.25 ± 0.02	1.33 ± 0.05	1.32 ± 0.03	1.26	0.31
Liver color	1.78 ± 0.28	1.89 ± 0.26	2 ± 0.24	0.19	0.83
Glycogen (mg/g liver)	18.95 ± 0.51 ^a^	22.89 ± 0.90 ^b^	29.50 ± 0.23 ^c^	76.13	0.00
Lipid content					
whole fish					
(g/100g, wet weight)	20.35 ± 0.39 ^a^	25.61 ± 0.38 ^b^	26.32 ± 1.24 ^b^	17.41	0.00
Adipose tissue					
(g/100g, wet weight)	74.48 ± 1.26 ^a^	78.75 ± 0.71 ^ab^	81.59 ± 1.54 ^b^	8.59	0.02
(g/tissue of a 100 g fish)	6.57 ± 0.51 ^a^	6.59 ± 0.32 ^a^	8.07 ± 0.22 ^b^	6.70	0.02
liver					
(g/100g, wet weight)	9.06 ± 1.01	8.65 ± 0.59	7.49 ± 0.24	1.41	0.29
(g/tissue of a 100 g fish)	0.13 ± 0.02	0.11 ± 0.01	0.1 ± 0.01	0.88	0.43
Muscle					
(g/100g, wet weight)	12.27 ± 0.39 ^a^	12.82 ± 0.17 ^ab^	13.85 ± 0.11 ^b^	10.09	0.01
(g/tissue of a 100 g fish)	7.8 ± 0.25 ^a^	8.12 ± 0.1 ^ab^	8.59 ± 0.07 ^b^	6.11	0.04

^1^ Values are means (n = 3). Values in a row that do not have the same superscript (a, b, c) are significantly different at *p* < 0.05 based on Tukey’s multiple range test. ^2^ Specific growth rate (SGR, %/day) = 100 × ln [final body weight (g)/initial body weight (g)]/days of the experiment. ^3^ Condition factor (CF) = 100 × [body weight (g)]/[body length(cm)]^3^. ^4^ Viscerosomatic index (VSI, %) = 100 × [viscera weight (g)]/[body weight (g)]. ^5^ Hepatosomatic index (HSI, %) = 100 × [hepatic weight (g)]/[body weight (g)].

**Table 4:** Plasma biochemical parameters of triploid rainbow trout fed the experimental diets for 77 days ^1^.

	Dietary Lipid Levels (% Dry Matter)			F	*p*
	20	25	30		
*Plasma biochemistry parameters*					
NEFA (umol/L) ^2^	418.27 ± 12.89 ^ab^	387.83 ± 12.45 ^a^	482.12 ± 19.73 ^b^	9.78	0.01
Glucose (mg/dL)	19.82 ± 0 ^a^	36.94 ± 0.9 ^ab^	46.4 ± 6.76 ^b^	11.72	0.04
Total cholesterol (mmol/L)	0.23 ± 0 ^a^	0.35 ± 0 ^b^	0.26 ± 0.03 ^a^	16.02	0.03
Triglyceride (mg/dL)	225.71 ± 17.5	262.26 ± 10.63	253.84 ± 9.3	2.17	0.26
HDL-C (mg/dL) ^3^	82.37 ± 1.55 ^a^	140.18 ± 14.11 ^b^	97.45 ± 8.12 ^ab^	10.08	0.05
LDL-C (mg/dL) ^4^	112.72 ± 0.19 ^a^	215.2 ± 4.45 ^b^	150.43 ± 18.17 ^a^	23.02	0.02

^1^ Values are means (n = 3). Values in a row that do not have the same superscript (a, b) are significantly different at *p* < 0.05 based on Tukey’s multiple range test. ^2^ NEFA: none-esterified fatty acid. ^3^ HDL-C: high-density lipoprotein-cholesterol. ^4^ LDL-C: low-density lipoprotein-cholesterol.

## Data Availability

The raw data supporting the conclusions of this article will be made available by the authors, without undue reservation. Data available on request due to restrictions eg privacy or ethical.
